# Insights in Immuno-Nutrition: Vitamin D as a Potent Immunomodulator

**DOI:** 10.3390/nu12113554

**Published:** 2020-11-20

**Authors:** Eva Untersmayr, Enikö Kallay

**Affiliations:** Institute of Pathophysiology and Allergy Research, Center for Pathophysiology, Infectiology and Immunology, Medical University of Vienna, 1090 Vienna, Austria; enikoe.kallay@meduniwien.ac.at

The relationship between nutrition and the immune system is a “complicated tango”, as coined earlier this year in a review in *Nutrients* [1]. There is growing amount of promising data indicating that selected nutrients beneficially modulate an ongoing exaggerated immune response in, e.g., autoimmunity or allergies, or support the immune system in the fight against pathogens. However, we still lack substantial evidence for a clear causality in controlled studies for most of them. Vitamin D is a nutrient with robust impact on the immune response, in addition to its classical function in calcium and phosphate homeostasis and bone metabolism. Vitamin D deficiency was linked to an increased inflammatory response in asthma and autoimmunity, such as in the case of rheumatic diseases.

A very interesting review article published in *Nutrients* in 2018 summarized the multiple effects of vitamin D on the immune response [2]. Francesca Sassi and co-authors highlighted the effects of the active form of vitamin D [1,25(OH)_2_D_3_ or calcitriol] on innate and adaptive immune cells, which express the vitamin D receptor. Moreover, immune cells are actively involved in vitamin D metabolism and modulation of local vitamin D concentration by converting 25(OH)D_3_ into 1,25(OH)_2_D_3_. Vitamin D is involved in the suppression of pro-inflammatory cytokines, modulating different T cell subtypes. It induces an overall tolerogenic immune response. Vitamin D plays a particularly central role in the context of the body’s microbiota composition and function, as well as in epithelial barrier function. As highlighted in the review, calcitriol contributes to enhanced barrier function, influences gut microbiota composition and reduces overall gut inflammation. This supports the concept of vitamin D as a booster of the immune defense against pathogens, which has gained prominence in recent months.

The global COVID-19 health crisis directed the focus of vitamin D research toward its impact on viral infections and host resistance, highlighting important new objectives for future studies. Based on the numerous studies suggesting that vitamin D has an influence on the antiviral immune response, it is reasonable to speculate that it could have a role in attenuating the symptoms of infections with the novel severe acute respiratory syndrome coronavirus 2 (SARS-CoV-2). Searching for the terms vitamin D and COVID-19 in PubMed in October 2020 resulted in 255 hits. Epidemiological evidence supports a therapeutic role of vitamin D in COVID-19 pathogenesis. A recent excellent review has summarized the antibacterial, antiviral, and anti-inflammatory actions of vitamin D, providing also an assessment on the link between vitamin D and COVID-19 [3]. Several studies found that vitamin D deficiency aggravated pneumonia and subsequent acute systemic inflammatory response syndrome [4], complications linked also to the pathogenesis of COVID-19. On the other hand, several preclinical and clinical studies found indication that vitamin D is effective in combating respiratory tract infections. Recently, vitamin D and quercetin were identified as candidate agents for mitigating the severity of COVID-19 [5]. However, results of different studies are contradictory, as one analysis suggested a negative correlation between vitamin D levels and the number of cases and deaths caused by COVID-19 in Europe [6]; while another group found no evidence for an association between COVID-19 transmission and UV radiation in 62 Chinese cities [7].

The association of vitamin D status with health outcome is well funded, and the evidence for a mechanistic role of vitamin D in regulating the immune system is convincing. However, the causality is still not clear. In a retrospective study, Radujkovic et al. [8] found an association between vitamin D deficiency and severity/mortality of COVID-19. A recent study by Pizzini et al. [9] in *Nutrients* showed that vitamin deficiency is common among COVID-19 patients, but a causal implication of vitamin D levels or vitamin D action on the disease course remained uncertain.

The conclusion of Sassi et al. is still valid: there is an urgent need for large-scale randomized controlled trials to confirm whether ensuring vitamin D sufficiency would reduce the incidence and severity of infections and/or autoimmune diseases.
